# Nutrient sensing and transceptor-mediated metabolic control in yeast

**DOI:** 10.1093/femsyr/foag027

**Published:** 2026-06-29

**Authors:** Ryoya Tanahashi, Hiroshi Takagi, Akira Nishimura

**Affiliations:** Department of Food Science and Technology, University of California, Davis, One Shields Avenue, Davis, CA 95616, United States; Strategic Initiative for Research and Innovation, Nara Institute of Science and Technology, 8916-5 Takayama-cho, Ikoma, Nara 630-0192, Japan; Strategic Initiative for Research and Innovation, Nara Institute of Science and Technology, 8916-5 Takayama-cho, Ikoma, Nara 630-0192, Japan; Faculty of Agriculture, Iwate University, 3-18-8, Ueda, Morioka, Iwate 020-8550, Japan

**Keywords:** nutrient sensing, nutrient signaling, PKA pathway, plasma-membrane transporters, *Saccharomyces cerevisiae*, transceptor

## Abstract

The yeast *Saccharomyces cerevisiae* coordinates growth, metabolism, and stress adaptation through signaling pathways that respond to changes in nutrient availability. Classical nutrient-sensing systems, including the cAMP–protein kinase A (PKA), Snf1/AMP-activated protein kinase, target of rapamycin complex 1 (TORC1)–Sch9, Ssy1–Ptr3–Ssy5, and general amino acid control pathways, have revealed how yeast senses and responds to extracellular carbon, nitrogen, phosphate, and amino acid levels. In addition to these established pathways, plasma-membrane nutrient transporters also function in signaling rather than solely mediating substrate uptake. These dual-function proteins, termed nutrient transceptors, couple nutrient transport or extracellular nutrient recognition to rapid intracellular responses, often activating PKA without detectable changes in cAMP levels. This review focuses on yeast nutrient transceptors, specifically Gap1, Mep2, Pho84, Sul1/Sul2, Can1, Ftr1, and Zrt1. These proteins link extracellular nutrient availability to intracellular regulatory responses, including trehalose mobilization, stress resistance, growth resumption, filamentous development, and, in some cases, TORC1–Sch9 signaling. Mechanistic insights, including transport-signaling uncoupling and potential physical association with downstream protein kinases, are also discussed. Collectively, this evidence establishes nutrient transceptors as an essential additional layer of nutrient sensing in yeast, highlighting their role in translating extracellular nutrient cues into cellular responses.

## Teaser

This review details nutrient transceptors in *Saccharomyces cerevisiae*, exploring how they link extracellular nutrient availability to intracellular signaling.

## Introduction

The yeast *Saccharomyces cerevisiae* plays a central role in industrial fermentations, including brewing and winemaking. It is responsible for both ethanol production and the formation of numerous flavor and aroma compounds in fermented products. Through carbon and nitrogen metabolism, *S. cerevisiae* converts simple substrates into a wide range of flavor-active molecules, including higher alcohols, esters, organic acids, and sulfur volatiles (Hazelwood et al. 2008). Extracellular carbon and nitrogen source availability and balance strongly influence these processes, shaping yeast physiology during fermentation (Crépin et al. 2012).

*Saccharomyces cerevisiae* has long served as a model organism for understanding how eukaryotic cells sense and respond to nutrient availability. Classical genetic and biochemical studies have identified a nutrient-sensing network that coordinates growth, metabolism, and stress resistance in response to changes in carbon, nitrogen, and other nutrients (Conrad et al. 2014, Rødkaer and Faergeman 2014). Major pathways in yeast respond to distinct nutrient classes. The cAMP/protein kinase A (PKA) pathway centrally regulates responses to fermentable carbon sources; the Snf1/AMP-activated protein kinase (AMPK) pathway is activated upon glucose depletion, relieving glucose repression (Santangelo 2006, Tamaki 2007, Hedbacker and Carlson 2008). These pathways coordinate transcriptional and metabolic shifts, enabling cells to transition from fermentative growth under high-glucose conditions to using alternative carbon sources. Concurrently, several other signaling systems primarily monitor nitrogen and amino acid availability. The target of rapamycin complex 1 (TORC1) integrates amino acid and overall nitrogen signals, promoting anabolic growth (González and Hall 2017). The SPS (Ssy1–Ptr3–Ssy5) system senses extracellular amino acids at the plasma membrane, while the general amino acid control (GAAC) pathway is activated during starvation via the Gcn2–Gcn4 system (Forsberg and Ljungdahl 2001, Conrad et al. 2014). These nitrogen-sensing pathways regulate protein synthesis, carbohydrate metabolism, stress defense, and cell-cycle progression in response to nitrogen status (Rubio-Texeira et al. 2010, Conrad et al. 2014).

Plasma-membrane proteins, including receptors and transporters, serve as the primary interface between the extracellular environment and intracellular metabolic networks. Nutrient transporters were long regarded as solely mediating substrate uptake. However, two decades of research shows that certain transporters, including those for amino acids, phosphate, and ammonium, also signal (Rubio-Texeira et al. 2010, Diallinas 2017, Steyfkens et al. 2018). In nutrient-starved cells, re-addition of a missing nutrient rapidly activates PKA in a cAMP-independent manner, promoting growth, mobilizing storage carbohydrates, and attenuating stress responses (Donaton et al. 2003, Giots et al. 2003, Van Nuland et al. 2006, Kankipati et al. 2015, Schothorst et al. 2017). Experiments with non-transported substrate analogs or transport-defective mutants further demonstrate that these proteins initiate signal transduction rather than merely mediating uptake (Popova et al. 2010, Van Zeebroeck et al. 2014, Kankipati et al. 2015). This work led to the concept of nutrient transceptors, dual-function proteins that combine transport activity with receptor-like signaling. Thus, the yeast nutrient-signaling network includes a distinct transceptor-dependent route to PKA activation (Thevelein et al. 2005, Rubio-Texeira et al. 2010).

This review first outlines the canonical nutrient-sensing pathways in *S. cerevisiae*, focusing on cAMP–PKA, Snf1/AMPK, TORC1–Sch9, SPS, and GAAC systems. We then discuss representative transceptors: the general amino acid permease Gap1, the phosphate transceptor Pho84, the sulfate transceptors Sul1 and Sul2, the ammonium transceptor Mep2, the arginine transceptor Can1, and the micronutrient transceptors Ftr1 and Zrt1. We further examine how these transceptors activate PKA and, in some cases, TORC1–Sch9 signaling, and explore how this activation influences metabolism, stress responses, and growth behavior. Finally, we evaluate the contribution of transceptors to yeast nutrient sensing, focusing on the intersection of transport, nutrient recognition, and intracellular regulation.

## Classical nutrient-sensing pathways

### The cAMP–PKA pathway

**Figure 1:**
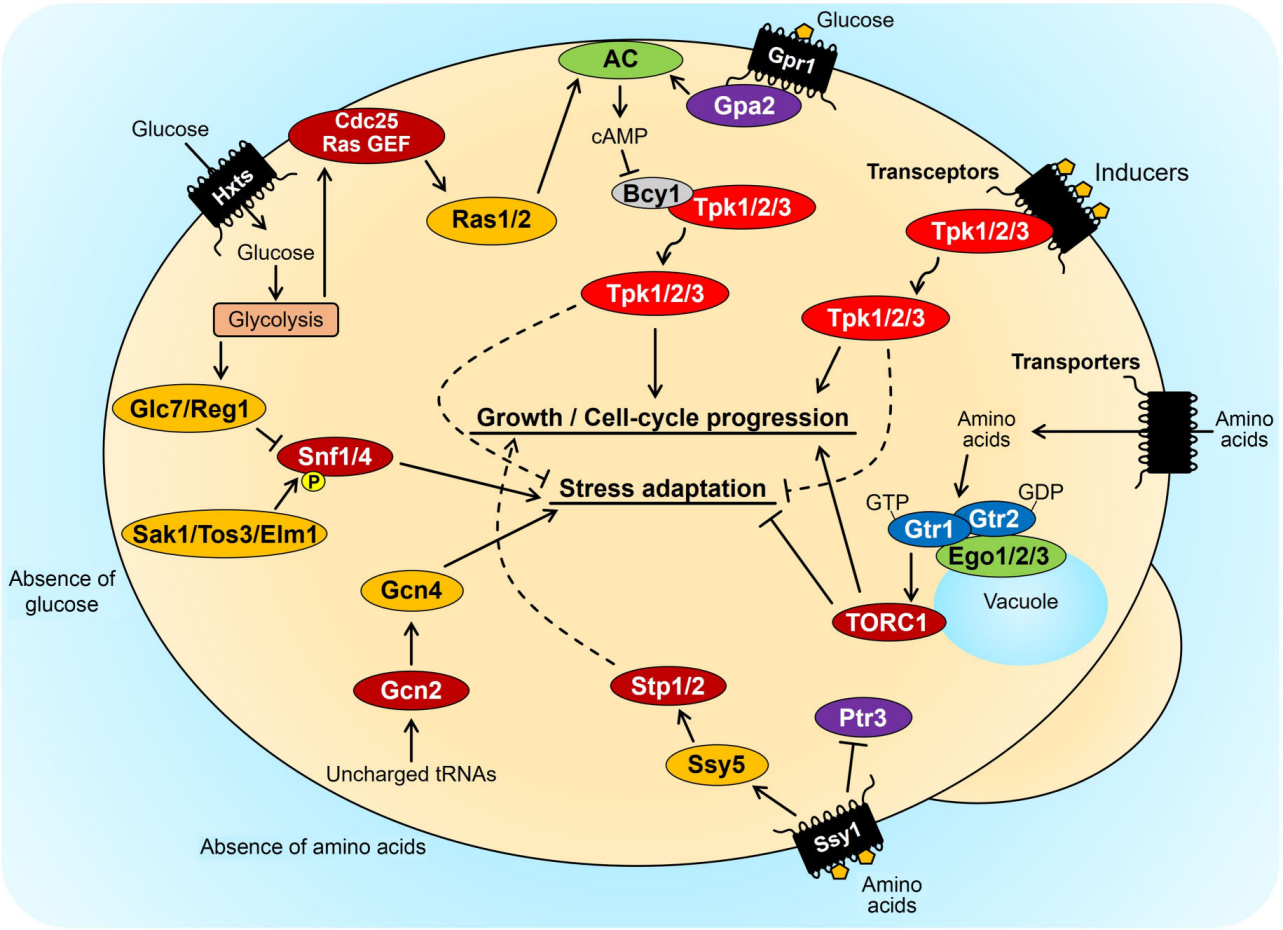
Overview of nutrient-sensing pathways in *Saccharomyces cerevisiae*. Yeast cells monitor nutrient availability through multiple signaling systems that coordinate growth and stress adaptation. Under glucose-rich conditions, glucose activates the cAMP–PKA pathway through two upstream inputs: extracellular glucose sensing via the G protein-coupled receptor Gpr1 and Gα subunit Gpa2, and intracellular glucose metabolism linked to the Cdc25–Ras system and adenylate cyclase (AC). The resulting increase in cAMP promotes binding of cAMP to the regulatory subunit Bcy1, leading to release and activation of the catalytic PKA subunits Tpk1, Tpk2, and Tpk3. Hexose transporters (Hxts) mediate glucose uptake, thereby supporting glycolytic input into this pathway. In contrast, glucose depletion activates the Snf1/AMPK pathway through the upstream kinases Sak1, Tos3, and Elm1, whereas glucose-rich conditions promote Reg1–Glc7-dependent dephosphorylation and inhibition of Snf1. Under glucose-limited conditions, Snf1/AMPK promotes stress adaptation through derepression of genes required for alternative carbon-source utilization. Amino acid starvation induces the general amino acid control (GAAC) pathway via Gcn2 and Gcn4 in response to uncharged tRNAs. The Gcn2–Gcn4 pathway promotes starvation responses and amino acid biosynthesis under amino acid limitation. Extracellular amino acids are sensed by the SPS system, in which Ssy1, Ptr3, and Ssy5 activate the transcription factors Stp1/2 and promote the expression of amino acid transporters. Intracellular amino acid availability also contributes to TORC1 activation through the active Gtr1-GTP/Gtr2-GDP state of the Rag GTPases associated with the EGO complex at the vacuolar membrane. In addition, nutrient transceptors act as a distinct sensing module that links nutrient repletion to rapid intracellular signaling, often converging on PKA-dependent responses. Together, these pathways form an interconnected network that regulates cellular outputs, including growth and cell-cycle progression and stress adaptation. Arrows indicate activation or positive regulation, whereas blunt T-bars indicate inhibition or negative regulation. P indicates phosphorylation.

The cAMP–PKA pathway is one of the best-characterized nutrient-signaling systems in yeast, linking glucose availability to rapid changes in metabolism, stress resistance, and proliferation (Santangelo 2006, Tamaki 2007). Glucose activates adenylate cyclase Cyr1 via two distinct upstream inputs: an extracellular sensing pathway involving the G protein-coupled receptor Gpr1 and G-alpha subunit Gpa2, and an intracellular pathway where the guanine nucleotide exchange factor Cdc25 and small GTPases Ras1 and Ras2 transmit signals from glucose catabolism and its metabolic intermediates (Fig. 1) (Thevelein and de Winde 1999, Santangelo 2006). The resulting increase in cAMP promotes binding of cAMP to the regulatory subunit Bcy1, leading to release of the catalytic subunits Tpk1, Tpk2, and Tpk3 and activation of PKA. Activated PKA phosphorylates targets involved in the mobilization of storage carbohydrates, such as the neutral trehalase Nth1, represses stress responses, and promotes cell-cycle progression (Thevelein and de Winde 1999, Conrad et al. 2014). PKA can also be activated by nutrient repletion in starved cells without a detectable rise in cAMP, a phenomenon central to transceptor signaling (Donaton et al. 2003, Giots et al. 2003, Van Nuland et al. 2006).

### TORC1 and Sch9

TORC1 centrally regulates cell growth, responding primarily to amino acid and nitrogen availability, with additional links to phosphate status (Conrad et al. 2014, González and Hall 2017). In *S. cerevisiae*, TORC1 regulation at the vacuolar membrane involves the EGO–Gtr GTPase system in response to amino acids, with Pib2 also contributing (Fig. 1). In this system, the Gtr1-GTP/Gtr2-GDP state promotes TORC1 activation, whereas the opposite Gtr1-GDP/Gtr2-GTP state is associated with reduced TORC1 activity (Dubouloz et al. 2005, Gao and Kaiser 2006, Varlakhanova et al. 2017, Ukai et al. 2018). Under nutrient-rich conditions, TORC1 promotes anabolic growth processes while repressing autophagy and stress responses. A major downstream effector of TORC1 is the AGC-family kinase Sch9, often regarded as a functional analog of mammalian S6 kinase (Conrad et al. 2014). TORC1-dependent phosphorylation of Sch9 stimulates ribosome biogenesis, protein synthesis, and cell-cycle progression, coupling biosynthetic activity to nutrient availability. While TORC1–Sch9 signaling has traditionally been viewed as distinct from the cAMP–PKA pathway, evidence reveals substantial functional overlap and coordination between these systems (Broach 2012, Conrad et al. 2014). This coordination is particularly important during nutrient refeeding, where rapid growth recovery relies on the integrated activation of multiple growth-promoting pathways rather than any single one.

### Snf1/AMPK, SPS, and GAAC

Beyond the cAMP–PKA and TORC1 pathways, *S. cerevisiae* employs several other nutrient-responsive systems that monitor distinct metabolic aspects. Activated upon glucose depletion, Snf1, the yeast ortholog of AMPK, plays a central role in adapting to carbon limitation (Hedbacker and Carlson 2008). Its activation depends on phosphorylation of the activation-loop residue Thr210 by the upstream kinases Sak1, Tos3, and Elm1 (Hedbacker and Carlson 2008, Liu et al. 2011). Conversely, under glucose-rich conditions, the Reg1–Glc7 phosphatase complex promotes dephosphorylation of Snf1 at the Thr210 residue, thereby suppressing genes for alternative carbon source utilization (Hedbacker and Carlson 2008). The SPS (Ssy1–Ptr3–Ssy5) system is a plasma-membrane pathway that senses extracellular amino acids. Amino acid sensing within the SPS system activates the endoprotease Ssy5, which processes the latent transcription factors Stp1 and Stp2. This proteolytic activation enables nuclear entry of Stp1 and Stp2, inducing genes for amino acid permeases (e.g. Agp1 and Bap2/3) and thereby enhancing amino acid uptake (Forsberg et al. 2001, Forsberg and Ljungdahl 2001, Ljungdahl and Daignan-Fornier 2012). By contrast, the GAAC pathway monitors intracellular amino acid starvation via uncharged tRNA accumulation. Gcn2 senses uncharged tRNAs, phosphorylating eIF2α to reduce global translation while promoting Gcn4 translation (Hinnebusch 2005, Conrad et al. 2014). Gcn4 subsequently induces genes for amino acid biosynthesis, nitrogen metabolism, and transport. Collectively, these systems enable yeast to adjust nutrient acquisition and metabolism based on carbon availability, extracellular amino acid supply, and intracellular amino acid status. These systems also integrate with the PKA and TORC1 pathways, which promote growth in nutrient-rich conditions but attenuate during carbon or nitrogen limitation (Fig. 1) (Broach 2012, Conrad et al. 2014).

## Nutrient transceptors

**Figure 2:**
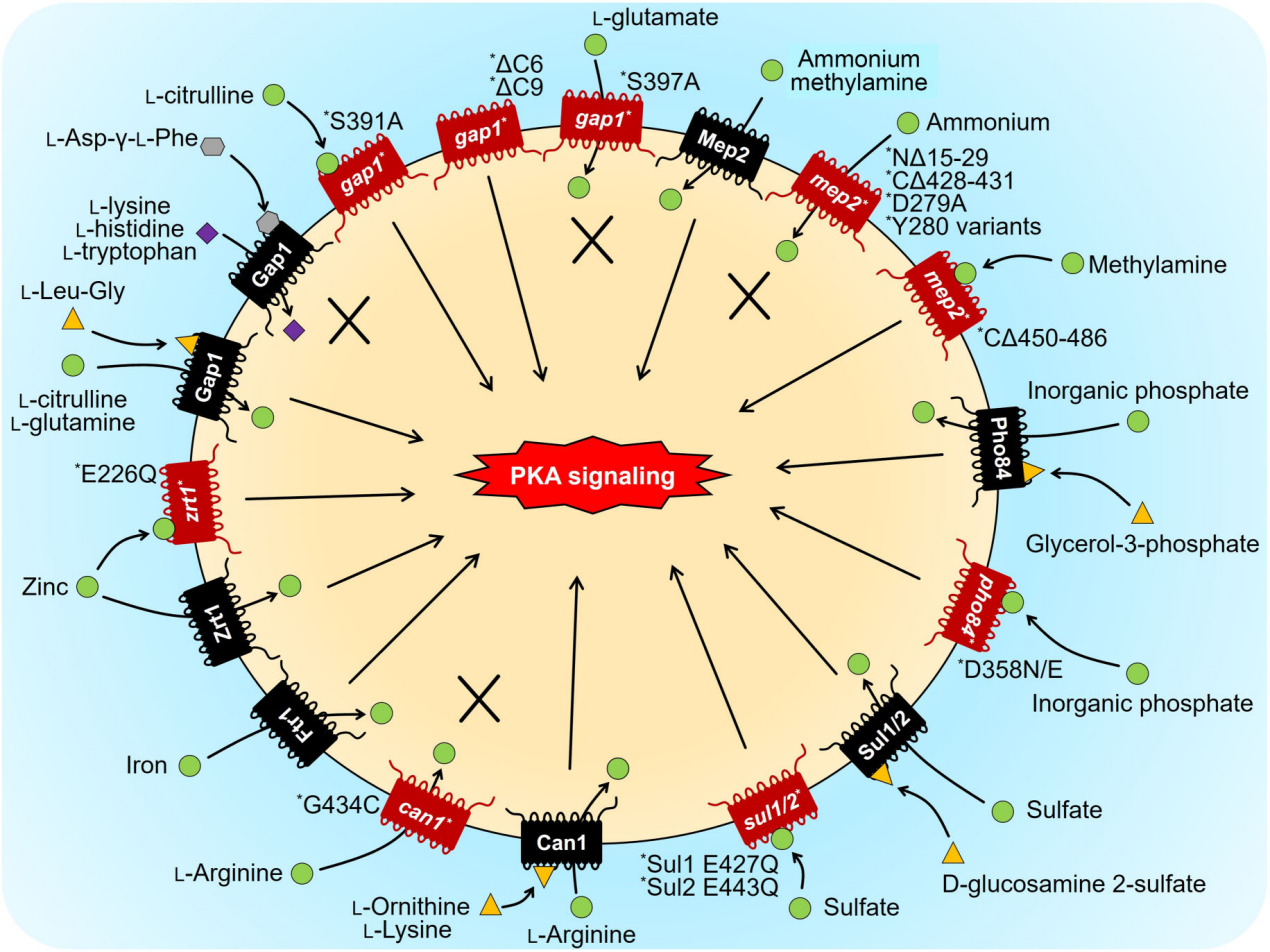
Representative substrates, ligands, and uncoupling mutants of yeast nutrient transceptors. The schematic summarizes representative nutrient transceptors described in Table 1 and their associated substrates, signaling agonists, non-signaling ligands, and transport-signaling uncoupling mutants. Green circles indicate transported substrates or ligands associated with transceptor-dependent signaling, yellow triangles indicate non-transported signaling agonists, purple diamonds indicate transported but non-signaling substrates, and gray hexagons indicate non-transported, non-signaling inhibitors. Black transporters represent wild-type transceptors, whereas red transceptors indicate representative uncoupling mutants in which nutrient transport and receptor-like signaling activities are functionally separated. Arrows toward the center indicate transceptor-dependent PKA signaling. Crosses indicate loss or absence of signaling under the indicated ligand or mutant condition.

Nutrient transceptors are dual-function membrane proteins that combine nutrient transport with receptor-like signaling (Rubio-Texeira et al. 2010, Diallinas 2017, Steyfkens et al. 2018). They are generally defined by three criteria: (i) mediation of nutrient transport under physiological conditions; (ii) necessity for rapid signaling responses, especially PKA pathway activation, upon nutrient repletion; and (iii) at least partial uncoupling of transport and signaling, demonstrable by substrate analogs or functional mutants (Rubio-Texeira et al. 2010, Schothorst et al. 2013). In *S. cerevisiae*, Schothorst et al. systematically identified the general amino acid permease Gap1, ammonium permease Mep2, phosphate transporter Pho84, and sulfate transporters Sul1/Sul2 as transceptors (Schothorst et al. 2013, Steyfkens et al. 2018). Subsequent studies extended this concept to additional transporters, such as the arginine transporter Can1 and micronutrient transporters (e.g. the high-affinity iron transporter Ftr1 and zinc transporter Zrt1) (Table 1 and Fig. 2) (Tanahashi et al. 2023a, Schothorst et al. 2017). Beyond yeast, (Steyfkens et al. 2018) proposed transceptors as evolutionary intermediates between nutrient transporters and dedicated receptors across eukaryotes (Diallinas 2017).

**Table 1:** Summary of representative nutrient transceptors in *Saccharomyces cerevisiae*.

Transceptor	Key substrates, ligands, and mutants	Readout	References
Gap1	L-citrulline; L-Leu-Gly (non-transported agonist); L-lysine/L-histidine/L-tryptophan (transported but non-signaling); L-Asp-γ-L-Phe (non-signaling inhibitor); S391A/S397A (uncoupling mutant); ΔC6/ΔC9 (constitutive active mutant)	Trehalase/PKA activation Trehalose/glycogen metabolism Reduced heat-shock resistance Repression of STRE genes	(Donaton et al. 2003, Van Zeebroeck et al. 2009, Van Zeebroeck et al. 2014)
Mep2	Ammonium; methylamine (transported but non-metabolizable analog); ΔN15-29/ΔC428-431/D279A/Y280 variants/ΔC450-486 (uncoupling mutant)	Trehalase/PKA activation; pseudohyphal/filamentous growth	(Van Nuland et al. 2006, Boeckstaens et al. 2008)
Pho84	Inorganic phosphate; glycerol-3-phosphate (non-transported agonist); D358N/E (uncoupling mutant)	Trehalase/PKA activation	(Popova et al. 2010, Samyn et al. 2012)
Sul1/2	Sulfate; D-glucosamine 2-sulfate (non-transported agonist); Sul1 E427Q/Sul2 E443Q (uncoupling mutant)	Trehalase/PKA activation Trehalose metabolism Replicative lifespan and stress signaling	(Kankipati et al. 2015, Long et al. 2025)
Can1	L-Arginine; L-Ornithine/L-Lysine (non-transported agonist); G434C (uncoupling mutant)	Trehalase/PKA activation Activation of Msn2/4-Shy1 cascade Put4 endocytosis Inhibition of proline utilization	(Tanahashi et al. 2023a, Nishimura et al. 2023, Tanahashi et al. 2025)
Ftr1	Iron	Trehalase/PKA activation	(Schothorst et al. 2017)
Zrt1	Zinc; E226Q (partial uncoupling)	Trehalase/PKA activation	(Schothorst et al. 2017)

Note: “Uncoupling mutant” refers to a mutant in which nutrient transport activity and signaling activity are functionally separated, allowing receptor-like signaling to be evaluated independently of substrate transport.

Many transceptors are strongly induced under starvation, a feature thought to prime cells for rapid responses upon nutrient repletion. Nutrient binding and/or transport is proposed to trigger conformational changes sensed by downstream signaling components, leading to rapid activation of PKA targets such as Nth1, often without a substantial increase in cAMP levels (Fig. 1) (Thevelein et al. 2005, Steyfkens et al. 2018). In some cases, transceptors also associate physically with Tpk1/2/3 or Sch9, suggesting a more direct link to growth-regulatory pathways (Zhang et al. 2021, Tanahashi et al. 2025).

## Amino acid and ammonium transceptors

### Gap1: a representative amino acid transceptor

The general amino acid permease Gap1 is strongly induced under nitrogen-poor conditions (Rubio-Texeira et al. 2010, Conrad et al. 2014). In nitrogen-starved cells, re-addition of selected amino acids rapidly activates PKA-dependent responses, such as Nth1 activation, reduced stress resistance, and trehalose degradation (Donaton et al. 2003, Van Zeebroeck et al. 2009). These responses depend on Gap1 and PKA’s catalytic subunits, yet occur without an increase in intracellular cAMP, indicating that Gap1 stimulates PKA via a cAMP-independent route. Further supporting Gap1’s transceptor classification, transport and signaling can be dissociated (Donaton et al. 2003, Van Zeebroeck et al. 2014). For example, peptides like L-Leu-Gly activate Gap1-dependent PKA signaling without being transported (Van Zeebroeck et al. 2009). Conversely, L-lysine, L-histidine, and L-tryptophan are transported but do not trigger PKA activation (Giots et al. 2003, Van Zeebroeck et al. 2009). In addition, mutations in Gap1’s cytosolic C-terminal tail uncouple signaling from transport, producing signaling-defective or constitutively active variants while preserving transport activity (Van Zeebroeck et al. 2014). Gap1-dependent PKA activation promotes trehalose breakdown via Nth1, suppresses Msn2/4-regulated stress-responsive gene expression, and supports rapid growth recovery after nitrogen repletion (Donaton et al. 2003, Van Zeebroeck et al. 2009, 2014). Gap1 thus remains a clear example of a nutrient transceptor, central to establishing the concept of amino acid-dependent, cAMP-independent PKA activation in yeast.

### Mep2: ammonium transceptor and morphogenetic switch

Mep2 is a high-affinity ammonium transporter with dual roles in nitrogen uptake and signaling. Deletion of *MEP2* severely impairs pseudohyphal growth under nitrogen-limiting conditions, while the loss of other ammonium transporters causes only modest defects (Lorenz and Heitman 1998). This phenotype suggests that the function of Mep2 extends beyond mere ammonium transport. Similar to Gap1, nitrogen-starved cells exhibit Mep2-dependent activation of PKA-related responses upon re-addition of ammonium or the non-metabolizable analog methylamine (Van Nuland et al. 2006). Genetic and epistasis analyses further implicate both the PKA pathway and a mitogen-activated protein kinase (MAPK) cascade, involving transcription factor Ste12, in Mep2 signaling (Lorenz and Heitman 1998, Rutherford et al. 2008). Through these pathways, Mep2 links nitrogen availability to morphogenetic programs, including pseudohyphal and filamentous growth. Although the underlying mechanism remains unresolved, structural and mutational studies suggest Mep2 adopts distinct conformational states for transport and signaling (van den Berg et al. 2016). Mep2 exemplifies how transceptor-mediated nutrient sensing regulates not only metabolic adaptation but also developmental responses to nutrient limitation.

## Phosphate and sulfate transceptors

### Pho84: a phosphate transporter linking PKA and TORC1

Pho84 is the major high-affinity phosphate transporter in *S. cerevisiae* (Giots et al. 2003, Mouillon and Persson 2006). During phosphate starvation, *PHO84* expression is strongly induced alongside Pho89, a Na⁺-dependent phosphate transporter (Mouillon and Persson 2006, Conrad et al. 2014). Upon phosphate repletion, Pho84 rapidly activates PKA targets, including trehalase, and mobilizes glycogen. Popova et al. and Samyn et al. demonstrated that certain phosphate analogs (e.g. glycerol-3-phosphate) act as signaling agonists without transport, and that specific mutations in Pho84, such as D358N/E and D178N/E, uncouple phosphate transport from PKA activation (Popova et al. 2010, Samyn et al. 2012). These findings classify Pho84 as a nutrient transceptor. Beyond PKA activation, Pho84 also regulates TORC1 activity. Loss or inhibition of Pho84 reduces TORC1 signaling and enhances cellular stress sensitivity, suggesting phosphate availability influences PKA- and TORC1-dependent growth control (Giots et al. 2003, Conrad et al. 2014, Zhang et al. 2021).

### Sul1 and Sul2: sulfate transceptors and lifespan control

Sul1 and Sul2 are high-affinity sulfate transporters strongly induced during sulfur starvation (Kankipati et al. 2015). In sulfur-starved cells, re-addition of sulfate rapidly activates PKA-dependent trehalase activity and other growth responses. These effects require Sul1 and Sul2 and occur with little or no increase in intracellular cAMP, indicating their function as sulfate transceptors. Beyond this acute signaling role, Sul1 is linked to cellular lifespan control (Long et al. 2025). Deletion of *SUL1* extends replicative lifespan, accompanied by altered PKA–Msn2-dependent stress signaling. This suggests that Sul1-dependent signaling, rather than sulfate transport alone, regulates stress resistance and longevity. Sul1 and Sul2 thus demonstrate that nutrient transceptors shape not only immediate metabolic and growth responses but also longer-term traits like lifespan.

## Arginine and micronutrient transceptors

### Can1: an arginine transporter that controls proline utilization

Can1, the principal arginine transporter in *S. cerevisiae*, was recently identified as a nutrient transceptor regulating proline utilization (Tanahashi et al. 2023a, Nishimura 2024). This function is particularly relevant to wine and beer fermentations, where inefficient proline consumption remains a persistent problem (Tanahashi et al. 2023b,c, Nishimura et al. 2022, 2024). Extracellular basic amino acids, including arginine, ornithine, and lysine, inhibit proline utilization by promoting endocytosis of the proline transporter Put4; the neutral amino acid citrulline does not elicit this effect (Nishimura et al. 2020a,b, Tanahashi et al. 2023a). This substrate specificity indicates that the response is not merely a consequence of nitrogen abundance but reflects a signaling mechanism sensitive to a specific group of extracellular amino acids. The involvement of ornithine and lysine, alongside arginine, further suggests that Can1-dependent regulation extends beyond its transport activity. Mutational analyses showed that arginine transport by Can1 itself is dispensable for arginine-mediated inhibition of proline utilization, supporting Can1’s role as a transceptor rather than a mere permease. Nishimura et al. subsequently demonstrated that activation of the Cdc25/Ras/cAMP–PKA pathway suppresses proline utilization under wine fermentation conditions (Nishimura et al. 2022). Loss-of-function mutations in *CDC25* alleviated this inhibitory phenotype, while artificial activation of PKA by dibutyryl-cAMP restored proline repression. These results position Can1-dependent control of proline metabolism within a broader PKA-linked signaling cascade.

Subsequent research clarified the downstream pathway. With PKA’s role established, later studies delineated a PKA–Msn2/4–Shy1 cascade that contributes to inhibition of proline utilization (Nishimura et al. 2023). Genetic analysis identified transcription factors Msn2 and Msn4 as functioning downstream of PKA. Shy1 also acts downstream of Msn2/4 and contributes to repressing proline utilization. In a commercial wine yeast strain *SHY1* deletion permitted proline consumption in a wine fermentation model without substantially affecting ethanol production, indicating that this pathway specifically restricts proline assimilation rather than overall fermentative capacity. More recent work revealed that Can1 physically associates with the PKA catalytic subunits Tpk1, Tpk2, and Tpk3, and identified a regulatory region within Can1 required for inhibiting proline utilization (Tanahashi et al. 2025). These observations support a direct signaling role for Can1 in controlling nitrogen source utilization.

Not all nutrient-dependent repression of proline utilization can be explained by the Can1 pathway alone. In a *can1Δ* strain, a reduced-function allele of *MET30* (encoding an SCF ubiquitin ligase component) was identified as an additional regulator of this phenotype (Nishimura et al. 2024). Genetic analysis indicated independent action of Met30 and Can1, suggesting that proline assimilation repression under nutrient-rich conditions involves at least two distinct pathways. The Met30-dependent branch showed inhibition when ammonium, methionine, or cysteine, and an additional amino acid (especially threonine or isoleucine), were present simultaneously. This pattern indicates a distinct, nutrient-responsive mechanism that parallels the Can1 transceptor pathway, reinforcing repression of proline utilization. Thus, Can1 functions as a nutrient transceptor, fine-tuning nitrogen source selection via PKA-linked signaling. Overall, proline metabolism in yeast is regulated by multiple, mechanistically distinct nutrient-responsive pathways.

### Ftr1 and Zrt1: micronutrient transceptors

Ftr1 and Zrt1, high-affinity transporters for iron and zinc, respectively, also function as micronutrient transceptors. Schothorst et al. identified them as transceptors by demonstrating that iron or zinc re-addition to nutrient-starved cells rapidly activates the PKA pathway in an Ftr1- or Zrt1-dependent manner (Schothorst et al. 2017). Later, Zhang et al. reported that Sch9 physically interacts with several nutrient transceptors (Zhang et al. 2021). This suggests a broader signaling network where micronutrient and macronutrient transceptors converge on PKA- and Sch9-mediated growth control. While Ftr1- and Zrt1-dependent signaling mechanisms are less defined than those for amino acid or phosphate transceptors, these studies expand the transceptor concept to micronutrient sensing. They further propose transceptor-mediated nutrient sensing as a general strategy for coupling diverse nutrient cues to growth-related signaling.

## Downstream signaling from nutrient transceptors

Nutrient transceptors frequently signal via the PKA pathway and its downstream effectors, enabling yeast cells to shift rapidly from stress-adapted states to renewed growth. A key downstream response is activation of the neutral trehalase Nth1 (Donaton et al. 2003, Giots et al. 2003, Van Nuland et al. 2006). PKA-dependent Nth1 phosphorylation promotes rapid trehalose degradation, supplying energy and carbon for growth resumption. Observed downstream of various macro- and micronutrient transceptors, trehalase activation is a common feature of nutrient-repletion signaling (Steyfkens et al. 2018). Another major consequence of transceptor-dependent PKA activation is the suppression of stress-responsive systems. Rim15, Msn2/4, and Gis1 centrally regulate quiescence, stress resistance, and longevity (Thevelein and de Winde 1999, Swinnen et al. 2006, Conrad et al. 2014). High PKA activity inhibits Rim15, excludes Msn2/4 from the nucleus, and represses stress-responsive gene expression (Santangelo 2006, Conrad et al. 2014). Conversely, reduced transceptor signaling lowers PKA activity, permitting reactivation of Rim15–Msn2/4-dependent stress responses and extending lifespan, as reported for cells lacking *SUL1* (Long et al. 2025). These observations demonstrate that nutrient transceptors influence not only acute metabolic adaptation, but also the broader balance between growth and stress resistance.

Beyond PKA-centered outputs, some studies suggest connections between nutrient transceptors and TORC1–Sch9-related growth control (Conrad et al. 2014, Zhang et al. 2021). Although evidence is more limited than for PKA, these observations indicate that extracellular nutrient sensing via transceptors may also converge on pathways governing ribosome biogenesis and other growth-associated transcriptional systems. Transceptor signaling can also extend beyond PKA-centered metabolic regulation. For instance, Mep2’s nutrient sensing feeds into a MAPK cascade that controls pseudohyphal differentiation, demonstrating transceptor influence on developmental decisions as well as metabolism (Lorenz and Heitman 1998, Rutherford et al. 2008). Overall, current evidence indicates that nutrient transceptors connect extracellular nutrient availability primarily to PKA-dependent outputs while also engaging TORC1–Sch9-related or MAPK signaling in some cases. This regulatory architecture allows yeast cells to transition efficiently from stress- and storage-oriented states to active growth, preserving nutrient-specific adaptations to distinct inputs such as ammonium, arginine, phosphate, and sulfate (Fig. 1).

## Concluding remarks and outlook

Studies on Gap1, Pho84, Sul1/Sul2, Mep2, Can1, Ftr1, and Zrt1 have established nutrient transceptors as a crucial component of nutrient sensing in *S. cerevisiae*. These proteins blur the conventional distinction between transporters and receptors by linking extracellular nutrient availability directly to intracellular growth-regulatory pathways, particularly PKA and TORC1–Sch9 signaling. Several important questions nevertheless remain. The molecular basis that distinguishes signaling from transport remains poorly understood. It also remains unclear how transceptor-derived signals integrate with Gpr1–cAMP signaling, metabolite-dependent inputs, and pathways involved in stress responses or development.

Nutrient transceptors are also attractive targets for applied research. By shaping how yeast responds to nutrient availability, they offer new opportunities for strain engineering in industrial fermentation, including controlling productivity, stress tolerance, and flavor-related traits. The implications of this field extend beyond yeast. As additional transceptors are identified in other eukaryotes, studies in *S. cerevisiae* will continue to inform broader principles of nutrient sensing. In higher organisms, where transporter-like proteins increasingly exhibit receptor-related functions, similar systems likely coordinate nutrient availability with growth and physiological regulation. Nutrient transceptors thus define a distinct regulatory layer that complements classical signaling pathways and contributes centrally to cellular adaptation to changing nutritional conditions.
